# A lesson from the ice bucket challenge: using social networks to publicize science

**DOI:** 10.3389/fgene.2014.00430

**Published:** 2014-12-15

**Authors:** Hashem Koohy, Behrad Koohy

**Affiliations:** ^1^Functional Genomics Group, Babraham Institute, University of CambridgeCambridge, UK; ^2^King Edward VI SchoolWarwickshire, UK

**Keywords:** amyotrophic lateral sclerosis, social network, Twitter, ice bucket challenge, ResearchGate, Mendeley, CiteULike

## 1. Background

Scientists are financially dependent on the general public. Salaries and research expenses come (directly or indirectly) from taxpayers. The public's understanding and appreciation of research is a motivational force for scientists. This is possibly one reason that scientists like their work to be covered by the media. However, scientists have shown very little success in more directly communicating with the public about their findings. Despite the fact that scientists have been very successful in solving many seemingly unsolvable problems, (Fermat Last Theorem, stepping onto the moon, sequencing genomes for many species, unraveling the complexity of different types of cancers, discovering the importance and applications of the stem cells, to name a few), they have shown little success in telling the public about the value and significance of these achievements nor have they made the public aware about existing challenges, leaving a big gap between scientific communities and the public. Given the ever-increasing popularity of social-network-based communications, scientists should ask themselves if they could use the potential of public Social Networks (SNs) for reducing this gap?

## 2. Social networks and scientists

SNs can be broadly divided into two types based on their applications: one type has a more specialized theme and aims for networking among specialists and focused communications. This group includes LinkedIn, ResearchGate, Mendeley, CiteULike and so on, which have gained some popularity among academics and other specific disciplines. For example, LinkedIn targets prospective employers and jobseekers in different fields. The second type however, has no specialized theme as such and has gained a universal popularity. Twitter and Facebook can be considered as the two key representatives of this group. Based on specialized theme of SNs in the first group, it is not surprising if we see scholars are biased toward the first group. However, for publicizing science, scientists should also care about the group in which the majority of the account holders are non-scientists.

As such, in what follows, we focus on the latter type of SNs, mainly Twitter, and first, try to illustrate their effectiveness and efficiency in communicating key issues to the public and then suggest a few ways of using Twitter with the ultimate aim of reducing the gap between scientists and the public.

## 3. Impact of SNs on our lives

SNs, in particular Twitter, have proven to be very powerful tools in changing political equations, social movements, marketing, business and permeate almost every part of our daily routines. One of the first instances we began to realize the power of Twitter was during the Green Movement Protests in Iran against the questionable presidential election in 2009. In a country with little Internet access the impact of using it was high enough to force authorities to react. Iranian authorities ordered it to be banned (its usage is still limited). The American Secretary of State, on the other hand, ordered their extra support and maintenance for that weekend. Similar usage of Twitter was then followed up in the Arab Springs and in the 2011 London Riots.

## 4. Publicizing science with SNs

During the past three months, two unplanned events initiated a great wave in SNs, giving rise to millions of pounds to be donated to cancer and ALS charities (see below). Without any doubt, they both increased the public awareness about these two—yet to be cured—diseases. The amount of money raised and the level of public awareness achieved for these two diseases were simply more than what the scientists had achieved over many years. The first which initiated on Facebook, was about teenage cancer and started when Steven Sutton put his story on Facebook and called for aid. The second was called Ice Bucket Challenge (IBC), and its ultimate aim was to combat Amyotrophic Lateral Sclerosis (ALS), a progressive neurodegenerative disease with 15 newly diagnosed patients per day. The life expectancy of diagnosed patients ranges between two to five years. However, thanks to the IBC, the public now knows more about this disease than ever before. In what follows, we aim to focus on ALS and IBC from a Twitter perspective, and illustrate the role of Twitter in directing the public attention to ALS. Although the origin of the IBC is not very clear, in particular, with the presence of a similar activity called “Cold Water Challenge,” it gained enormous popularity in SNs during July and August 2014 when millions of people took part in this activity.

**Figure 1:**
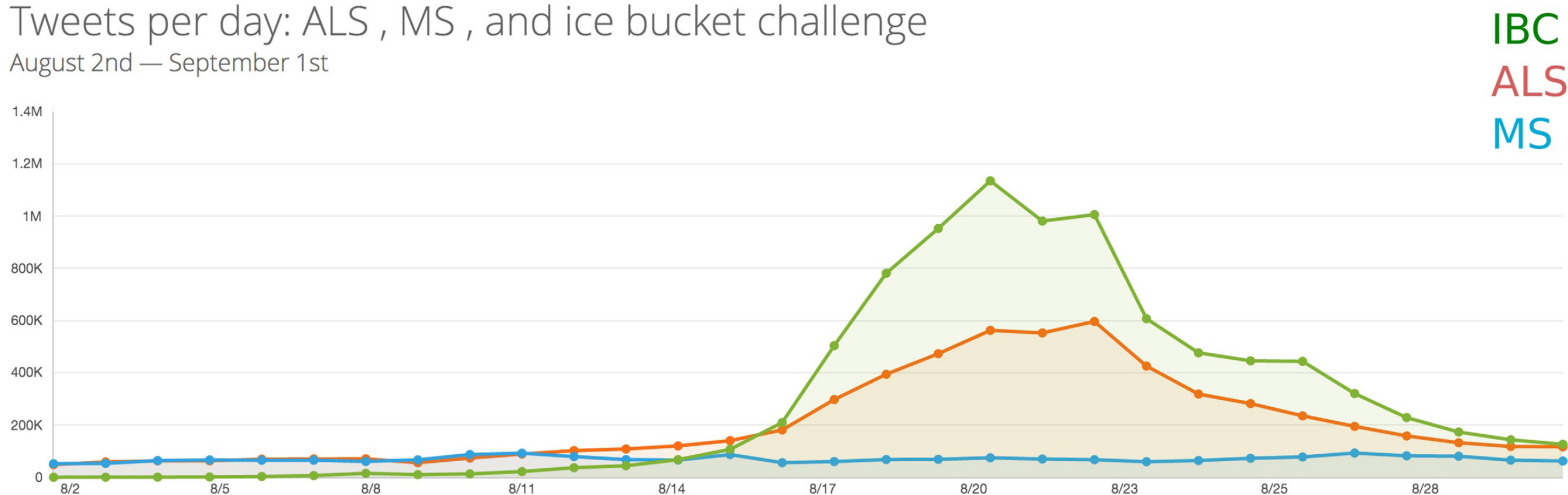
**Illustrated here is the number of tweets of #ALS, #MS and #Icebucketchallange on a daily basis (y-axis) during August 2014 (x-axis)**.

The participants included people from various backgrounds, ranging from high profile celebrities and politicians, to people from the poorest regions of the globe with limited access to internet and SNs. The initial idea of the IBC was to pour cold water on one's head with the aim of raising money for charities working on ALS-related subjects. Besides raising an incredible amount of funds (US $98.2m, from the beginning to 29 of August 2014, which is 35 times more than the same period in last year) IBC has remarkably helped prompt the public awareness about one of the Twenty first century's challenges in science. To further illustrate this, we have compared the daily number of tweets of #icebucketchallenge vs. #ALS during August 2014. We have also considered the daily number of #MS (Multiple Sclerosis) tweets as a control. Results are shown in Figure 1, which shows how Twitter-based discussion about IBC and ALS are related.

Interestingly, during the third week of the August, when millions of people were tweeting about IBC, hundreds of thousands of people on a daily basis were tweeting about ALS. This is considerably greater than the number of people tweeting about Multiple Sclerosis (MS), a more wide-spread and known disease to the public. Although this figure is based on Twitter's data, we obtained a very similar pattern from the Google trends when we compared the number of searches of IBC vs. “what is ALS?”. More statistics and data about the IBC challenge can be found at Shuck (2014).

The huge success of the IBC has already attracted some attention from different disciplines (Boko, 2014; Tucker, 2014) and surely will be the topic of more sophisticated studies in the future. Nevertheless, it provides an example in which the power of SNs was enhanced by clearly defining a task in a simple yet fun way, with a valuable incentive, promoting a good feeling amongst participants. In addition, the task almost exponentially spread across SNs, since each participant had to challenge two new people to take part.

We agree that this level of success in using SNs for a particular task is not easy to achieve. Nevertheless, an increased usage of SNs is a key step in engaging public awareness.

It is hard to obtain an accurate measure of the fraction of scientists actively using Twitter, but it is estimated to be only 2.5% (Priem, 2011; Darling et al., 2013). We therefore recommend the non-users, in particular, those who are hesitant, to pay more attention to the power of these SNs. It is worth noting that SNs are advancing very quickly, in 5 years time it might be harder to join in. Also, there is always the possibility that SNs will loose their popularity (for some unforeseen reason) and those who haven't joined might miss the chance to reach others by these means.

We are not aware of any systematic study on why scientists show less interest in Twitter and Facebook. However, based on personal discussions three reasons are frequently mentioned: (a) They do not see any point of using it, (b) They are worried about unsecured status of these SNs, in particular, about revealing the details of their private life and unpublished scientific work, (c) They are worried about being bombarded with noise-like feeds.

We agree that these problems and risks are likely for the twitter users. However, (a) We hope that our example illustrates the functionality of Twitter for scientific communications, (b) There are a few actions that can be taken to increase the security of your account. In particular the beginners are recommended to follow the twitter security and safety guidelines (see Twitter, 2014) (c) By choosing your followers and your followings, in fact, you are directing the kind of discussions you would like to take part than can prevent - at least to some extent- from being bombarded by unwanted tweets.

Once you have started your virtual socialization, based on your interests, which are reflected in your feeds, posts and connections, you are likely to be followed by a limited number of your science colleagues, science students, journalists, politicians and so on. However, according to the six degrees of separation rule (Wikipedia, 2014), your tweet can be read by any other user (billions for Twitter) in fewer than six re-tweets. This provides you with a fantastic opportunity to present your science.

In the remainder of this section we suggest a number of specific things that you can start with.

As a postgraduate student or a junior scientist you can:

- Live-tweet the scientific conferences that you attend. This is indeed a good way of learning more about talks and posters as you try to tweet their key messages in 140 characters. For a useful guide we refer the reader to Ekins and Perlstein (2014).
- Discuss recently published papers. This encourages you to understand each paper thoroughly. You are also very likely to learn new facts during these informal discussions that you may have overlooked. This eventually helps good science to be appreciated and bad science to be filtered out.
- Connect with the leading figures in your field. This keeps you updated, not only in terms of scientific advances in your field, but also of potential suitable jobs being offered in these groups.
- Be interactive. Discuss the challenges you face and ask your followers' pinions about them. Believe it or not, there is always someone out there to assist you. Also, do not be shy in expressing your opinion about other tweets and/or questions that are asked.
- If your are new to the field of computational biology then it is worth noting that companies like Google, Twitter and Facebook are pioneers in developing super fast searching models, memory management, data storage, handling big data and visualizations which are also some fundamental issues in computational biology. So being active in tweeter and other SNs might put you in a position to think about some of underlying machine learning techniques that fascinate you.

And for the senior scientists, further to what have we suggested for the junior scientists:

- Highlight the importance of your findings to the public.
- Make your science understandable. Your papers published in high impact journals are often far too difficult to be appreciated by the majority. So, why not highlight their key messages in plain English.
- Broaden your expertise by discussing your science with researchers from other disciplines.
- Engage directly with the public rather than having the media distracting your scientific messages. Remember how the incredible amount of work and discoveries in the ENCODE project were distracted by inappropriate presentation of only one of its scientific messages (see Gregory, 2012).
- Advertise job opportunities in your group. In particular, young people have shown more active in SNs, so this increases your chance of attracting undergraduate and postgraduate candidates.

## 5. Conclusions

SNs are fast developing and becoming extremely popular. They have proven very influential and able to change various aspects of our lives. They can be very instrumental for the scientific communities for further outreaching and educating the public about our scientific discoveries. The design and popularity of Twitter has made it a very loud micro blog and, to some extent, unique for further public engagement with scientific activities. For further discussion about this, please follow me on Twitter: @hashemkoohy.

## Author contributions

Hashem Koohy: conceived, designed, analyzed and wrote, Behrad Koohy: exported data, analyzed and wrote.

### Conflict of interest statement

The review editor John Hancock declares that, despite being affiliated at the same institution as the author Hashem Koohy, the review process was handled objectively and no conflict of interest exists. The authors declare that the research was conducted in the absence of any commercial or financial relationships that could be construed as a potential conflict of interest.

## References

[B1] Boko G. (2014). 6 Viral-Marketing Lessons to Learn from the Ice Bucket Challenge. Available online at: http://www.entrepreneur.com/article/236843

[B2] Darling E. S. Shiffman D. Cote I. M. Drew J. A. (2013). The role of twitter in the life cycle of a scientific publication. PeerJ, 1, 1–31 10.7287/peerj.preprints.16v1

[B3] Ekins S. Perlstein E. O. (2014). Ten simple rules of live tweeting at scientific conferences. PLOS Comput. Biol. 10:e1003789. 10.1371/journal.pcbi.1003789 25144683 PMC4140634

[B4] Gregory T. R. (2012). The Encode Media Hype Machine. Available online at: http://www.genomicron.evolverzone.com/2012/09/the-encode-media-hype-machine/

[B5] Priem J. (2011). As Scholars Undertake a Great Migration to Online Publishing, Altmetrics Stands to Provide an Academic Measurement of Twitter and Other Online Activity. Available online at: http://blogs.lse.ac.uk/impactofsocialsciences/2011/11/21/altmetrics-twitter/

[B6] Shuck J. (2014). The Data Behind the Ice Bucket Challenge. Available online at: http://www.plentyconsulting.com/ice-bucket-challenge-data

[B7] Tucker C. (2014). Why the Ice Bucket Challenge Proved Such a Runaway Success. Available online at: https://www.yahoo.com/tech/why-the-ice-bucket-challenge-proved-such-a-runaway-100262238434.html

[B8] Twitter H. C. (2014). Stay Safe and in Control of your Twitter Experience. Available online at: https://support.twitter.com/groups/57-safety-security

[B9] Wikipedia. (2014). Six Degrees of Separation — Wikipedia, the Free Encyclopedia. Available online at: http://en.wikipedia.org/w/index.php?title=Six_degrees_of_separation&oldid=623070045

